# Supplementary material for: Spatial transcriptomics map of the embryonic mouse brain – a tool to explore neurogenesis
Source: Biol Open. 2023 Oct 19;12(10):bio060151. doi: 10.1242/bio.060151 (PMC10602001; doi:10.1242/bio.060151)
Supplement: Supplementary information [file biolopen-12-060151-s1.pdf]

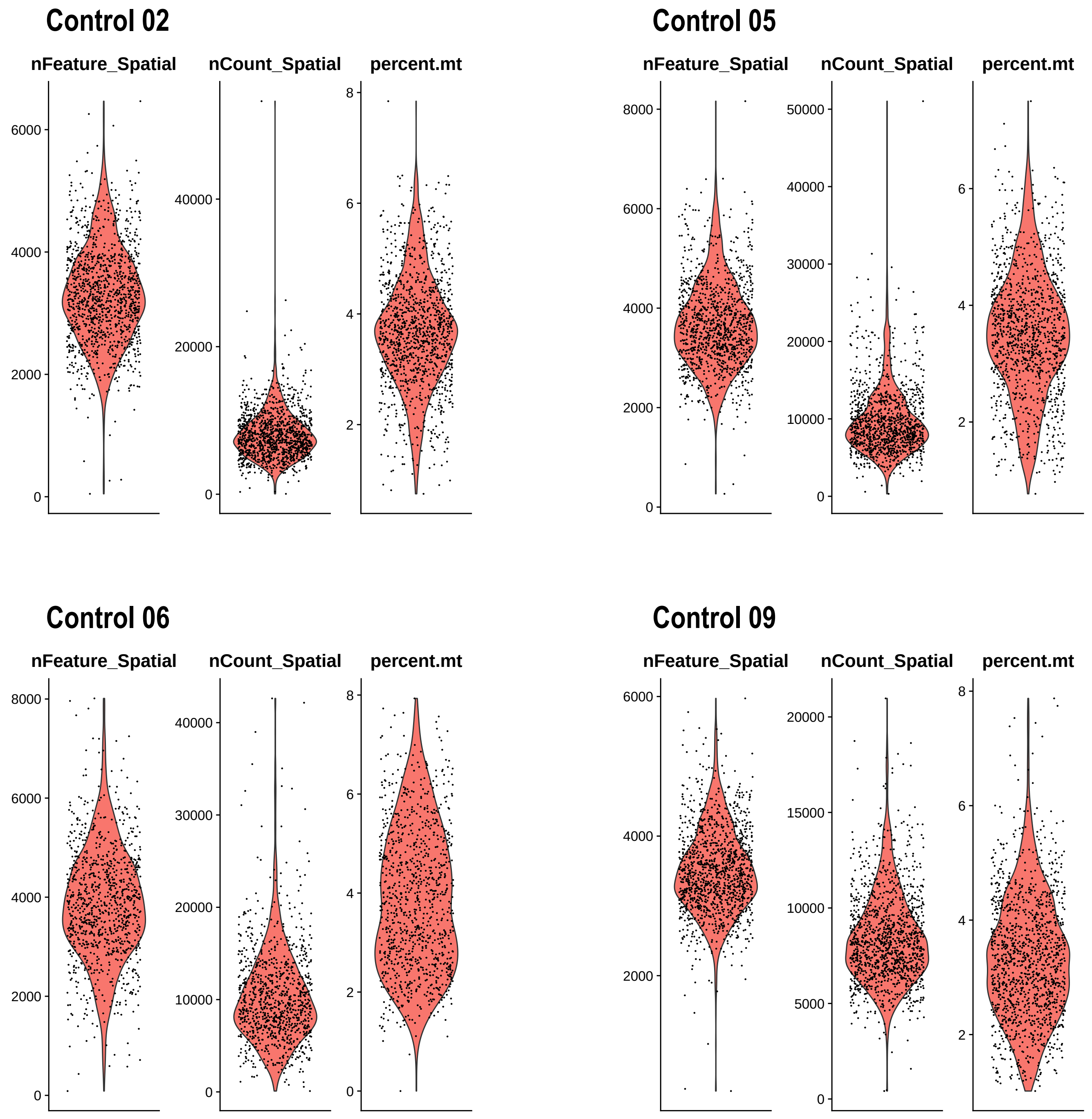

Fig. S1. Spatial Transcriptomics QC metric for the E13.5 mouse embryonic sections used in this study

Biology Open • Supplementary information

control_02

Alerts

The analysis detected ⚠ 1 warning.

	Alert	Value	Detail
⚠	Low Fraction Reads in Spots	48.7%	Ideal >50%. Application performance may be affected. Many of the reads were not assigned to tissue covered spots. This could be caused by high levels of ambient RNA resulting from inefficient permeabilization, because the incorrect image was used, or because of poor tissue detection. The latter case can be addressed by using the manual tissue selection option through Loupe.

Summary | Analysis

1,196
Number of Spots Under Tissue

75,268
Mean Reads per Spot

3,262
Median Genes per Spot

Sequencing

Number of Reads	90,020,988
Valid Barcodes	96.4%
Valid UMIs	100.0%
Sequencing Saturation	69.4%
Q30 Bases in Barcode	93.0%
Q30 Bases in RNA Read	91.4%
Q30 Bases in UMI	92.8%

Mapping

Reads Mapped to Genome	93.1%
Reads Mapped Confidently to Genome	87.8%
Reads Mapped Confidently to Intergenic Regions	5.3%
Reads Mapped Confidently to Intronic Regions	6.8%
Reads Mapped Confidently to Exonic Regions	75.6%
Reads Mapped Confidently to Transcriptome	73.7%
Reads Mapped Antisense to Gene	1.0%

Spots

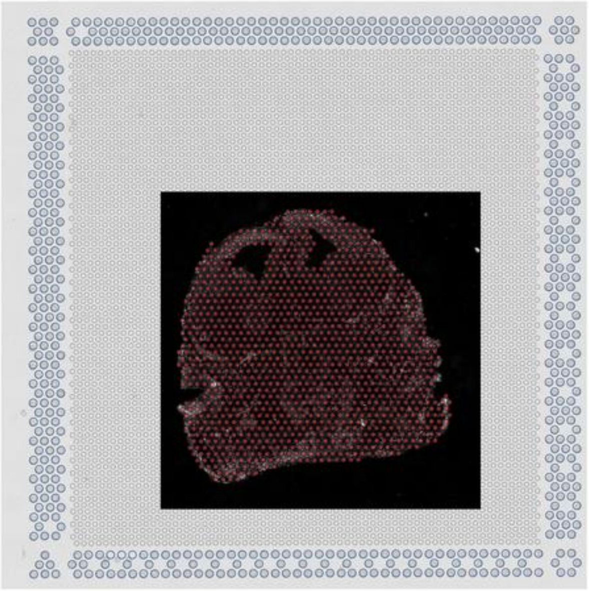

Fraction Reads in Spots Under Tissue	48.7%
Mean Reads per Spot	75,268
Mean Reads Under Tissue per Spot	33,352
Median UMI Counts per Spot	7,554
Median Genes per Spot	3,262
Total Genes Detected	19,056

Sample

Sample ID	control_02
Sample Description	
Chemistry	Spatial 3' v1
Slide Serial Number	V10N16-037-A1
Reference Path	.../omics/odcf/analysis/OE0446_projects
Transcriptome	mm10-2020-A
Pipeline Version	spaceranger-1.3.0
Image Reorientation	Off

Biology Open • Supplementary information

Biology Open (2023): doi:10.1242/bio.060151: Supplementary information

control_05

Summary Analysis

1,152
Number of Spots Under Tissue

91,653
Mean Reads per Spot

3,588
Median Genes per Spot

Sequencing

Number of Reads	105,584,760
Valid Barcodes	96.3%
Valid UMIs	100.0%
Sequencing Saturation	70.5%
Q30Bases in Barcode	92.9%
Q30Bases in RNA Read	90.7%
Q30Bases in UMI	92.7%

Mapping

Reads Mapped to Genome	92.4%
Reads Mapped Confidently to Genome	86.7%
Reads Mapped Confidently to Intergenic Regions	5.9%
Reads Mapped Confidently to Intronic Regions	6.3%
Reads Mapped Confidently to Exonic Regions	74.5%
Reads Mapped Confidently to Transcriptome	72.6%
Reads Mapped Antisense to Gene	1.0%

Spots

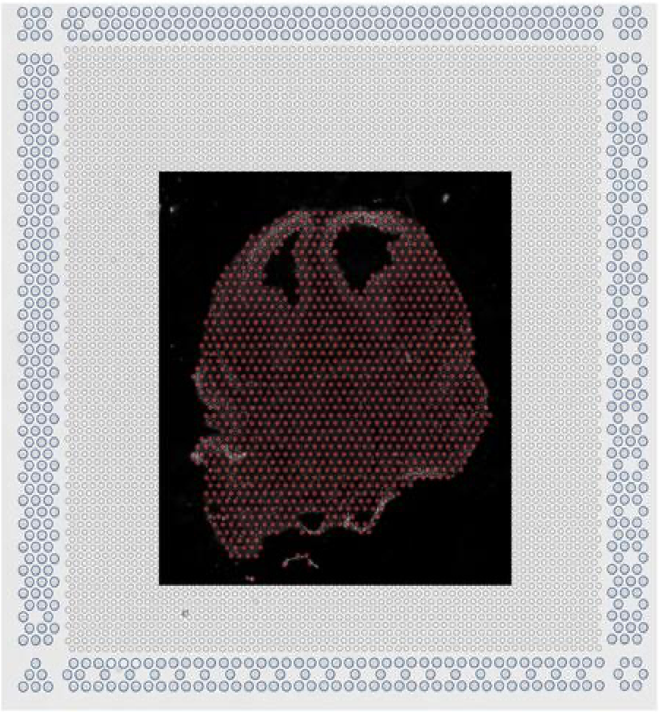

Fraction Reads in Spots Under Tissue	52.4%
Mean Reads per Spot	91,653
Mean Reads Under Tissue per Spot	43,791
Median UMI Counts per Spot	8,797
Median Genes per Spot	3,588
Total Genes Detected	19,163

Sample

Sample ID	control_05
Sample Description	
Chemistry	Spatial 3' v1
Slide Serial Number	V10N16-037-B1
Reference Path	.../omics/odcf/analysis/OE0446_projects
Transcriptome	mm10-2020-A
Pipeline Version	spaceranger-1.3.0
Image Reorientation	Off

Biology Open • Supplementary information

control_06

Alerts

The analysis detected ⚠ 1 warning.

	Alert	Value	Detail
⚠	Low Fraction Reads in Spots	46.4%	Ideal >50%. Application performance may be affected. Many of the reads were not assigned to tissue covered spots. This could be caused by high levels of ambient RNA resulting from inefficient permeabilization, because the incorrect image was used, or because of poor tissue detection. The latter case can be addressed by using the manual tissue selection option through Loupe.

Summary Analysis

984
Number of Spots Under Tissue

105,113
Mean Reads per Spot

3,815
Median Genes per Spot

Sequencing

Number of Reads	103,431,356
Valid Barcodes	96.2%
Valid UMIs	100.0%
Sequencing Saturation	70.8%
Q30 Bases in Barcode	92.9%
Q30 Bases in RNA Read	91.4%
Q30 Bases in UMI	92.7%

Mapping

Reads Mapped to Genome	93.7%
Reads Mapped Confidently to Genome	88.4%
Reads Mapped Confidently to Intergenic Regions	5.1%
Reads Mapped Confidently to Intronic Regions	5.6%
Reads Mapped Confidently to Exonic Regions	77.7%
Reads Mapped Confidently to Transcriptome	75.8%
Reads Mapped Antisense to Gene	1.0%

Spots

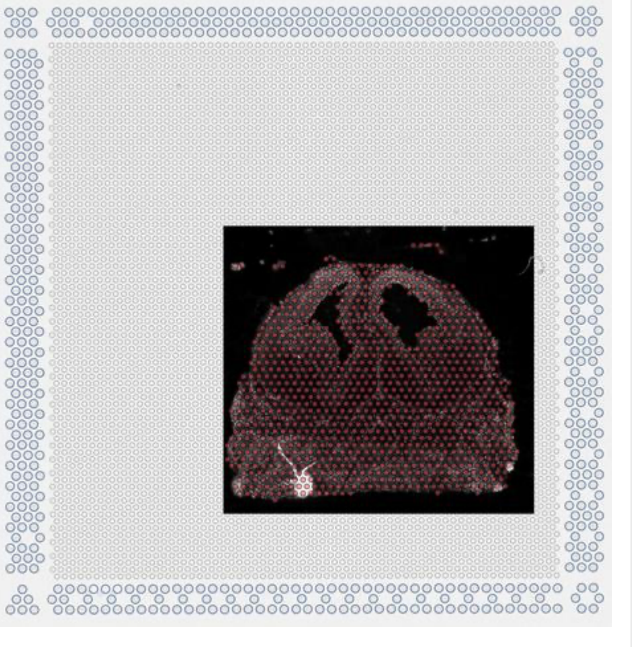

Fraction Reads in Spots Under Tissue	46.4%
Mean Reads per Spot	105,113
Mean Reads Under Tissue per Spot	44,454
Median UMI Counts per Spot	9,496
Median Genes per Spot	3,815
Total Genes Detected	18,991

Sample

Sample ID	control_06
Sample Description	
Chemistry	Spatial 3' v1
Slide Serial Number	V10N16-041-C1
Reference Path	…/omics/odcf/analysis/OE0446_projects
Transcriptome	mm10-2020-A
Pipeline Version	spaceranger-1.3.0
Image Reorientation	Off

Biology Open • Supplementary information

control_09

Summary | Analysis

1,266
Number of Spots Under Tissue

61,676
Mean Reads per Spot

3,436
Median Genes per Spot

Sequencing

Number of Reads	78,081,917
Valid Barcodes	96.5%
Valid UMIs	100.0%
Sequencing Saturation	61.9%
Q30 Bases in Barcode	92.6%
Q30 Bases in RNA Read	90.9%
Q30 Bases in UMI	92.4%

Mapping

Reads Mapped to Genome	91.7%
Reads Mapped Confidently to Genome	85.3%
Reads Mapped Confidently to Intergenic Regions	6.8%
Reads Mapped Confidently to Intronic Regions	7.4%
Reads Mapped Confidently to Exonic Regions	71.0%
Reads Mapped Confidently to Transcriptome	69.5%
Reads Mapped Antisense to Gene	0.6%

Spots

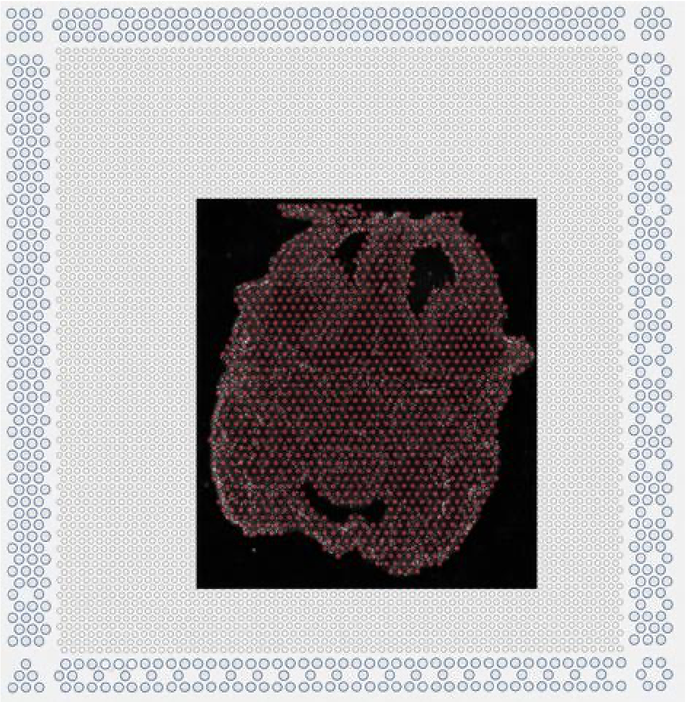

Fraction Reads in Spots Under Tissue	54.1%
Mean Reads per Spot	61,676
Mean Reads Under Tissue per Spot	29,250
Median UMI Counts per Spot	8,124
Median Genes per Spot	3,436
Total Genes Detected	19,030

Sample

Sample ID	control_09
Sample Description	
Chemistry	Spatial 3' v1
Slide Serial Number	V10N16-041-D1
Reference Path	…/omics/odcf/analysis/OE0446_projects
Transcriptome	mm10-2020-A
Pipeline Version	spaceranger-1.3.0
Image Reorientation	Off

Biology Open • Supplementary information

Table S1.

Available for download at
https://journals.biologists.com/jcs/article-lookup/doi/10.1242/bio.060151#supplementary-data

Table S2.

Available for download at
https://journals.biologists.com/jcs/article-lookup/doi/10.1242/bio.060151#supplementary-data

Table S3.

Available for download at
https://journals.biologists.com/jcs/article-lookup/doi/10.1242/bio.060151#supplementary-data

Biology Open • Supplementary information
